# Adolescent experiences, perceptions, and preferences for the process of HIV status disclosure in Kenya

**DOI:** 10.3389/fpubh.2023.1165557

**Published:** 2023-12-01

**Authors:** Cyrus Mugo, Irene N. Njuguna, Kristin Beima-Sofie, Caren W. Mburu, Alvin Onyango, Janet Itindi, Barbra A. Richardson, Laura Oyiengo, Grace John-Stewart, Dalton C. Wamalwa

**Affiliations:** ^1^Kenyatta National Hospital, Research and Programs, Nairobi, Kenya; ^2^Department of Epidemiology, University of Washington, Seattle, WA, United States; ^3^Department of Global Health, University of Washington, Seattle, WA, United States; ^4^Department of Pediatrics and Child Health, University of Nairobi, Nairobi, Kenya; ^5^Kenya Medical Research Institute, Kisumu, Kenya; ^6^Department of Biostatistics, University of Washington, Seattle, WA, United States; ^7^National AIDS and STI Control Program, Ministry of Health, Kenya, Nairobi, Kenya; ^8^Department of Medicine, University of Washington, Seattle, WA, United States; ^9^Department of Pediatrics, University of Washington, Seattle, WA, United States

**Keywords:** disclosure, human immunodeficiency virus (HIV), adolescents and youth, Kenya, satisfaction, caregiver, healthcare worker, provider

## Abstract

**Introduction:**

Disclosure of HIV status to adolescents living with HIV has been associated with improved treatment outcomes. However, there are limited data regarding the experiences of, perceptions of, and preferences for the process of disclosure of HIV status among adolescents and young adults living with HIV (AYLH), especially in sub-Saharan Africa.

**Methods:**

Young adults living with HIV from 20 HIV clinics in Kenya who participated in a clinical trial evaluating the effectiveness of a disclosure and transition package completed an anonymous survey in 2019. We described their experiences and preferences using counts and proportions and assessed factors associated with satisfaction with the disclosure process using linear regression, reporting age-adjusted mean differences (aMD), and 95% confidence intervals (95%CIs).

**Results:**

Of the 375 enrolled AYLH, 265 (71%) had perinatally acquired HIV, of whom 162 (61%) were female. The median age of the enrolled AYLH was 16 years (IQR: 14–19 years), and all of them were on antiretroviral therapy (ART). For over half (55%) of the participants, caregivers disclosed their HIV status, and 57% preferred that their caregivers disclose the status to them. Most (78%) of the participants preferred full disclosure by 12 years of age. The majority (69%) believed the disclosure was planned, and 11% suspected being HIV positive before the disclosure. Overall, 198 (75%) AYLH reported that they were ready for disclosure when it happened, and 86% were satisfied with the process. During both pre-disclosure (67 and 70%, respectively) and post-disclosure (>75% for each), AYLH felt supported by the clinic and caregivers. Factors associated with higher satisfaction with the disclosure process were pre-disclosure clinic support (aMD: 0.19 [95%CI: 0.05–0.33]) and pre-disclosure (aMD: 0.19 [0.06–0.31]) and post-disclosure (aMD: 0.17 [0.03–0.31]) caregiver support. AYLH who suspected they were HIV positive before they were disclosed to tended to have lower satisfaction when compared to those who never suspected (aMD: −0.37 [−0.74-(−0.01)]). Overall, they reported that disclosure positively influenced their ART adherence (78%), clinic attendance (45%), and communication with caregivers (20%), and 40% reported being happier after disclosure.

**Conclusion:**

Young adults living with HIV advocated for an appropriately timed disclosure process with the involvement of caregivers and healthcare workers (HCWs). Support from caregivers and HCWs before and during disclosure is key to improving their disclosure experience.

## Introduction

Adolescents and young adults living with HIV (AYLH) are a growing population in sub-Saharan Africa (SSA) (1). The increase in the proportion of AYLH is partly due to the transition of a large population of survivors of perinatally transmitted HIV into adolescence following improved access to antiretroviral treatment (ART) in the last decade (2). In addition, adolescents and young adults aged 15–24 years have higher HIV incidence when compared to other age groups (1). Despite improved treatment options and implementation of adolescent-friendly care, AYLH have poorer viral suppression and high HIV-related mortality when compared to adults (3). Dose adjustment challenges and the development of resistance to medications contribute to this disparity (4). In addition, poor outcomes result from poor adherence to treatment, stemming from factors including inappropriate HIV status disclosure (e.g., unintentional disclosure to an adolescent before they are prepared over time to receive information about their status); a living situation that limits, for example, where the adolescent can store medication; and school schedules that are barriers to clinic attendance. HIV stigma and negative attitudes and beliefs about people with HIV are major barriers, and one example of the latter is when the adolescent anticipates that being seen taking medications may result in them being discriminated against. Other factors include adolescent rebellion (the adolescent defies guidance from authorities, in this case, on treatment adherence and clinic attendance) and poor mental health (5–8).

Disclosure of HIV status to adolescents who acquired HIV perinatally has been associated with improved retention in care, ART adherence, viral suppression, and mental health (9, 10). From a public health perspective, disclosure before a sexual debut improves the chances of preventing future transmission (4). Challenges affecting the disclosure process in SSA include caregivers’ reluctance to disclose (11), poor access to appropriate disclosure tools and materials that guide providers and caregivers on the process of disclosure (12), and a heavy workload among healthcare workers (HCWs) in facilities (13). The World Health Organization (WHO) guidelines recommend that children advance through a planned and structured disclosure process (14). Various tools have been developed to guide the process for AYLH in SSA (15, 16). Recommended disclosure practices include conducting disclosure for children between the ages of 6 and 12 years and involving both caregivers and HCWs in the process (14). However, in SSA, full disclosure typically occurs at a later age, with 50% taking place after the age of 13 years (9, 17, 18). The involvement of caregivers is also sub-optimal due to fears related to their competency to lead the process and their ability to support the emotional needs of the child after disclosure (18, 19). Studies examining the impact of lower age of disclosure and caregiver involvement in the disclosure process on adolescent adherence and disease progression show a positive impact (20).

While multiple studies have examined HCW and caregiver preferences to inform guidelines on 128 disclosure (18), fewer studies have examined adolescent perspectives on these practices. Understanding the adolescent perspective on the disclosure process is essential for improving the disclosure experience and incorporating patient-centered care approaches. Two qualitative studies in Southern Africa show that AYLH prefer early disclosure performed in a healthcare setting (21, 22). More research is required to understand these preferences in different contexts, how these preferences affect AYLH’s satisfaction, and the outcome of the disclosure process. In this study, we determined AYLH’s preferences on when disclosure should happen, who should be involved, and whether they felt prepared and supported to know their status and their satisfaction with the disclosure process. Furthermore, we determined AYLH’s perception of the impact disclosure had on their adherence, clinic attendance, sexual behavior, and subsequent self-disclosure to others.

## Materials and methods

### Study design

This cross-sectional analysis is nested in the Adolescent Transition to Adult Care for Adolescents Living with HIV in Kenya (ATTACH) study, a cluster randomized clinical trial (RCT; NCT03574129) assessing the impact of an adolescent transition package developed in Kenya on transition outcomes for AYLH (23). Data for this analysis were collected from AYLH aged 12–24 years who were enrolled in 20 large HIV clinics in four of 47 counties in Kenya between October and November 2019, prior to the RCT. The clinics did not have standard disclosure practices addressing differences, especially of age, at full disclosure, the involvement of caregivers and providers in the disclosure process, and the utilization of disclosure tools described in a previous study (24).

### Theoretical framework

**Figure 1:**
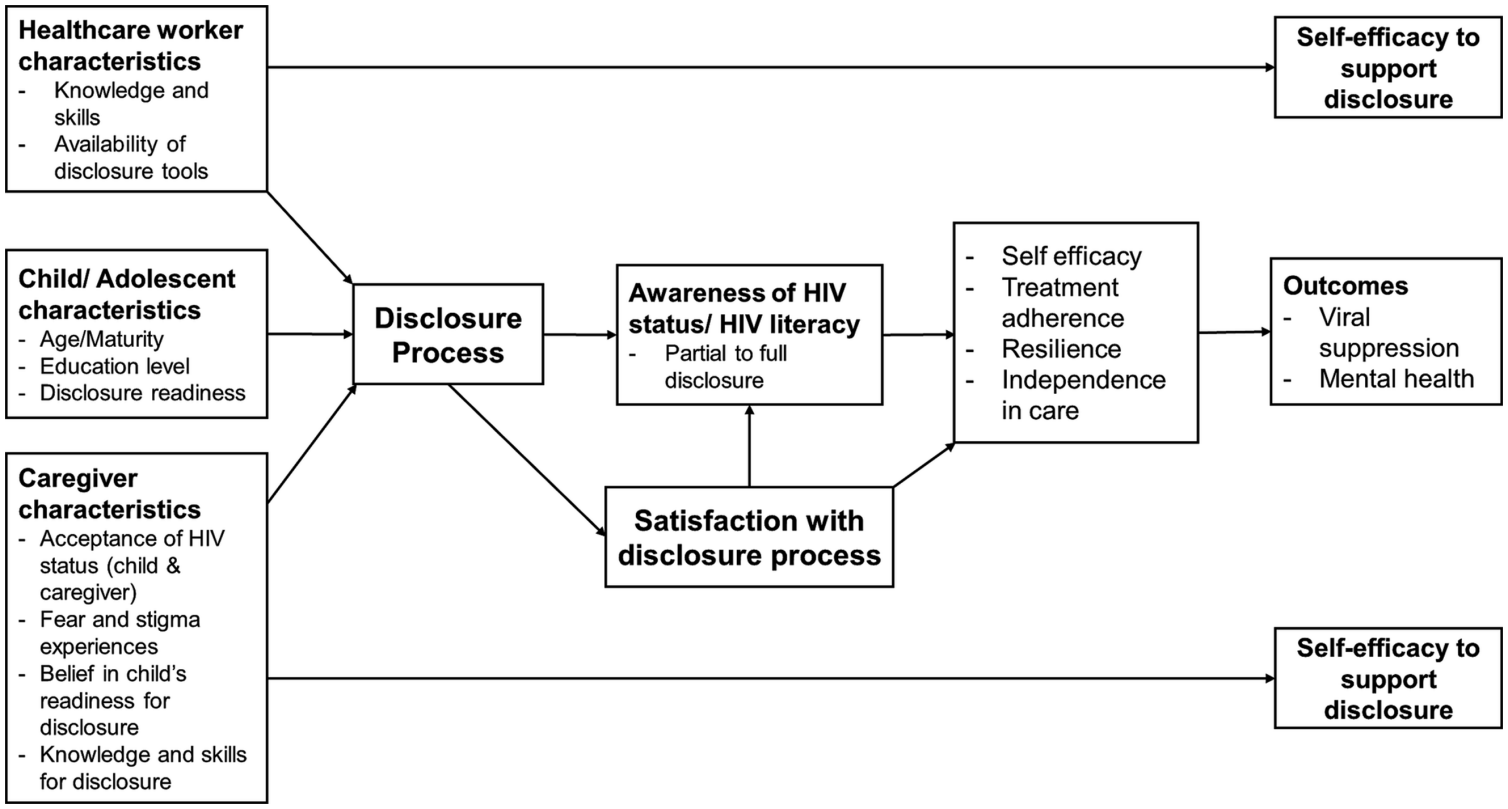
Theoretical model of HIV status disclosure to children and adolescents.

This study is based on a theoretical disclosure framework (Figure 1) adapted from an earlier study by O'Malley et al. (25). This framework is broad and captures the three key stakeholders in a disclosure process to AYLH—the adolescent, their caregiver, and the healthcare worker. Characteristics of these key stakeholders that may affect the disclosure process and modify its effect on treatment, mental health, and other outcomes include the following: (1) the age and education level of the child; (2) caregiver’s own acceptance of the child’s and, in some cases, their HIV status, HIV stigma, their belief on when a child is ready to be disclosed to, and the skills they have to guide the disclosure process; and (3) the healthcare worker’s skills to guide the disclosure process and the tools at their disposal to guide them, the caregiver, and the child in the disclosure process. In previous studies where this framework was applied, provider and caregiver self-efficacy to disclose were assessed. In this study, we proposed to add a key factor—AYLH’s satisfaction with the disclosure process—and theorized that it mediates the effect of the disclosure process on desired outcomes. We also hypothesized that satisfaction with the disclosure process is influenced by the characteristics of AYLH, caregivers, and HCWs described above. We tested the latter relationships in this study and describe the proposed outcomes from the perspective of AYLH. In future studies, we aim to assess the impact of satisfaction on the proposed outcomes.

### Participant recruitment

The study recruited AYLH visiting the HIV clinic. The recruiting study nurse screened AYLH attending the clinic to ascertain their age, disclosure status, and willingness to participate in the study. Those who were aged 12–24 years and confirmed that they received HIV care (i.e., were fully disclosed to) provided oral consent to complete an anonymous survey. We recruited a convenient sample of at most 20 AYLH per clinic.

### Data collection and measures

The study nurses conducted a 30-min survey with each AYLH after their routine clinic visit in a private space within the clinic. Surveys were administered using a tablet with the REDCap mobile app already installed, with the nurse reading the question aloud, offering clarifications to the participant if needed and recording the participant’s response. The data were stored and managed using the secure web-based REDCap platform hosted at the University of Washington, Seattle, WA, United States. Sociodemographic information, including the age of the participant, sex, education level, county of residence, and living companion (the person they lived with), was collected. We also collected HIV-related information, including duration since the start of ART and length of time since they were disclosed to.

To describe their disclosure experience, AYLH provided information on who disclosed their HIV status to them (i.e., caregiver, HCW, self-discovery, or underwent HIV testing to know their status), whether they perceived that the disclosure was planned, and whether they already suspected they had HIV before disclosure was done. To assess disclosure readiness, they were asked whether they felt prepared to receive the information about their HIV status at the time of disclosure (yes, no, or not sure). They also rated the support they received from HCWs and caregivers on Likert scales before disclosure (always/high support, usually, sometimes, or never) and after disclosure (a lot/high support, some, a little, or not at all). Satisfaction was assessed using one question asking AYLH to rate their satisfaction with the disclosure process on a Likert scale (not satisfied, somewhat satisfied, satisfied, or very satisfied/high satisfaction).

We assessed AYLH’s preferences for the disclosure process with the following questions: Who do you think should tell children that they have HIV? (caregiver, HCW, or do not know); at what age should children living with HIV be told that they have HIV? (open-ended); and when should your disclosure have happened? (earlier, later, never, or do not know). Finally, we assessed the AYLH’s perceived impact of disclosure on sexual behavior, adherence, and clinic attendance. AYLH were asked: Do you feel that knowing you have HIV helped you? If yes, how was it helpful? Choices included the following: taking medication on time, abstaining from sex, practicing safer sex, talking freely with healthcare workers, and communicating better with the caregivers.

### Ethical considerations

The ATTACH study was approved by the institutional review board (IRB) of the University of Washington and the ethics and research committee (ERC) of the University of Nairobi and the Kenyatta National Hospital, Nairobi, Kenya. To conduct the anonymous pre-trial surveys, the IRB and ERC waived parental consent for minors (12–17 years) since the majority were unaccompanied to the clinic, and the study required that they knew their HIV status to be enrolled. The survey was also considered low risk, with participants only required to provide oral consent. Since no personal identifying information was collected from participants, a breach of confidentiality was of higher risk with written documentation rather than verbal agreement. In cases where a caregiver accompanied an adolescent to the clinic, the study respected their wish if they did not want their child to complete the anonymous survey.

### Analysis

In this analysis, we included AYLH who acquired HIV perinatally. We defined perinatally acquired HIV as meeting any one of the following three criteria: (1) currently aged 15 years or younger, (2) disclosure was done by a caregiver or a family member, or (3) being on ART before the age of 15 years. Descriptive statistics (counts and proportions) were used to characterize sociodemographic characteristics, disclosure practices, preferences, and impact of disclosure. Disclosure practices included who disclosed (caregiver, HCW, or adolescent figured out themselves), whether the disclosure was planned, and whether the AYLH suspected being HIV positive before disclosure. AYLH’s disclosure preferences included the preferred time for disclosure (classified as earlier, later, never, or do not know) and preferred person to disclose (caregiver, HCW, or other persons).

Linear regression was used to assess factors associated with satisfaction with the disclosure process by AYLH. Satisfaction was included in the model as a continuous outcome variable, converting the Likert scores as follows: 4, very satisfied; 3, satisfied; 2, somewhat satisfied; and 1, not satisfied.

We conducted univariate analyses with the following independent variables: age, sex, education level, duration on ART, duration since the disclosure, preferred time for disclosure, disclosure readiness, the person involved in disclosure, whether the disclosure was planned, and suspicion of HIV status before disclosure. Preparation by clinic and clinic and caregiver support before and after disclosure were included as continuous variables after their Likert scales were converted to numeric scales. We then included age in all the models as a confounder. Age was adjusted for due to differences in understanding of the disclosure process by adolescents at different ages (21). We accounted for clustering within the facility in both the unadjusted and age-adjusted analyses by deriving 95% confidence intervals (95%CIs) for the mean differences from bootstrapped t-statistics and reported bootstrapped values of *p* (26).

#### Power considerations

A convenience sample of 250–350 AYLV was adequate to describe the prevalence of key disclosure components, i.e., full disclosure by the age of 12 years, disclosure by a caregiver, and whether there was a plan to disclose, with a precision of 2–3%. With this sample, we had a power of greater than 0.8 to detect differences of at least 0.5 in the mean satisfaction levels for predictor variables with a prevalence of 0.5–0.9. We used R Studio (Version 1.1.456, 2009–2018) for the analyses.

## Results

### Description of participants

Of the 375 enrolled AYLH, 265 (71%) were classified as having perinatally acquired HIV, and 156 (59%) were from regions with an adult (≥15 years) HIV prevalence ≥5%. Of the enrolled AYLH, 162 (61%) belonged to the female sex, and the median age was 16 years (IQR: 14–19 years). All AYLH were on ART. Of the enrolled AYLH, 234 (88%) reported ART use for 5 or more years, and 293 (78%) were living with their family. Overall, 174 (66%) reported knowing their HIV status for 5 years or more (Table 1).

**Table 1:** Sociodemographic characteristics and HIV treatment history of adolescents.

Characteristic, *N* = 265	*n* (%)
*County of residence (HIV prevalence)*	
Homa Bay (Hyper-endemic [>11%])	77 (29)
Nairobi (High prevalence >5 and < 11%)	79 (30)
Nakuru (Low–medium prevalence <5%)	46 (17)
Kajiado (Low–medium prevalence <5%)	63 (24)
*Female sex*	162 (61)
*Age*	
12–14	49 (18)
15–19	129 (49)
20–24	87 (33)
*Education level*	
Primary	128 (48)
Secondary	128 (48)
Tertiary	9 (4 s)
*Duration on ART*	
<1 year	7 (3)
1–4 years	23 (9)
≥5 years	234 (88)
*Living companion (Person they lived with)*	
Parent	170 (64)
Other family (grandparent/uncle/aunt/sibling)	69 (26)
Partner	14 (5)
Other (lives alone/friends)	12 (5)
*Duration since disclosure*	
<1 year	17 (11)
1–4 years	72 (37)
≥5 years	174 (66)

Lower total number (*N*) for some variables due to missing responses.

### Disclosure practices and preferences

The majority of AYLH were informed of their status by a caregiver (145 [55%]) or an HCW (108 [41%]). Only 15 (4%) AYLH discovered their positive HIV status on their own. The majority (200 [78%]) preferred disclosure to occur by 12 years of age. Over half (138 [57%]) of them preferred that caregivers disclose, 97 (40%) preferred disclosure by HCWs, and 6 (3%) preferred disclosure done by a family member. We assessed preferences for the person disclosing according to age. Among the younger AYLH (age groups 12–14 and 15–19 years), 53% preferred disclosure by a caregiver, while among the older group (20–24 years), 72% preferred disclosure by a caregiver (*p* < 0.023). The majority (166 [69%]) of ALYH believed that the disclosure was planned. Only a few, however (30 [11%]), suspected that they were HIV-positive before disclosure (Table 2).

**Table 2:** Disclosure practices, support, preparation, and outcomes.

Characteristic (*N* = 265)	*n* (%)
*Who disclosed HIV status*	
Caregiver	145 (55)
Healthcare worker	108 (41)
Adolescent figured out	15 (4)
*Who should disclose*	
Caregiver	138 (57)
Healthcare worker	97 (40)
Other persons	6 (3)
*Preferred age for disclosure*	
<10 years	52 (20)
10–12 years	148 (58)
13–15 years	42 (17)
>15 years	13 (5)
*Preferred time for disclosure*	
Earlier	131 (51)
Later	43 (17)
Never	9 (3)
Do not know	74 (29)
*Planned disclosure*	
Yes	166 (69)
No	43 (18)
Do not know	31 (13)
*Adolescent suspected being HIV positive*	
Yes	30 (11)
No	227 (86)
Do not know	7 (3)
*Clinic prepared adolescent*	
A lot	134 (54)
Some	52 (21)
A little	45 (18)
Not at all	18 (7)
*Pre-disclosure clinic support*	
Always	169 (67)
Usually	33 (13)
Sometimes	29 (12)
Never	19 (8)
*Pre-disclosure caregiver support*	
Always	174 (70)
Usually	23 (9)
Sometimes	27 (11)
Never	26 (10)
*Post-disclosure support by the clinic*	
A lot	212 (80)
Some	29 (11)
A little	21 (8)
Not at all	2 (1)
*Post-disclosure support by the caregiver*	
A lot	209 (79)
Some	17 (7)
A little	29 (11)
Not at all	8 (3)
*Was ready to be disclosed to*	
Yes	198 (75)
No	60 (23)
Not sure	7 (2)
*Satisfaction with disclosure*	
Very satisfied	77 (34)
Satisfied	119 (52)
Somewhat satisfied	15 (6)
Not satisfied	18 (8)
*How disclosure was helpful	
Adolescents happier after disclosure	106 (40)
Improved communication with healthcare worker	68 (26)
Improved communication with caregiver	54 (20)
Disclosure helped me abstain from sex	41 (15)
Disclosure helped me practice safer sex	34 (13)
Disclosure improved adherence	207 (78)
Disclosure improved clinic attendance	118 (45)
*Self-disclosure	
To anyone	124 (47)
To family	97 (37)
To friends	26 (10)
To intimate partners	24 (9)

Lower total number (*N*) for some variables due to missing responses. *Non-exclusive categories.

**Figure 2:**
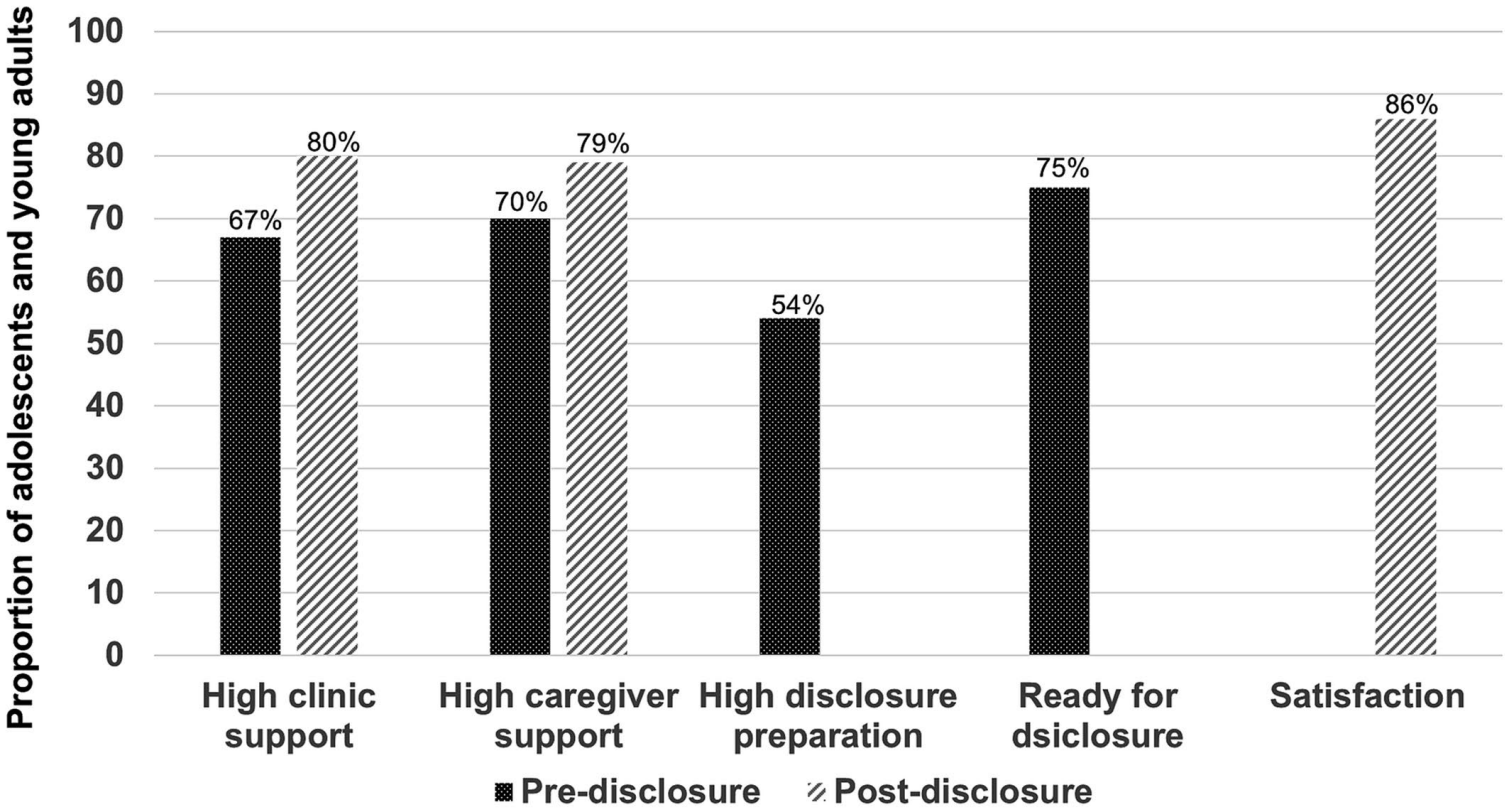
Perceived disclosure support, preparation, readiness, and satisfaction.

Overall, 134 (54%) AYLH felt that the clinic prepared them adequately for disclosure, and 198 (75%) were ready for disclosure when it took place. Among the 60 AYLH who were not ready for disclosure, 43 (72%) preferred that disclosure was done later, 9 (15%) preferred that they never were disclosed to, while 8 (13%) said they did not know whether the timing of disclosure was appropriate. Over two-thirds felt supported adequately by the clinic and caregiver before disclosure (67 and 70%, respectively), while over three-quarters felt supported adequately after disclosure by the clinic and caregiver (80 and 79%, respectively; Figure 2).

### Satisfaction with the disclosure process and disclosure outcomes

Participants reported their satisfaction with the disclosure process, with 196 (86%) AYLH reporting being satisfied or very satisfied. AYLH reported multiple ways the disclosure impacted them, with 207 (78%) reporting improved medication adherence, 118 (45%) reporting improved clinic attendance after disclosure, and 106 (40%) reporting that they were happier after disclosure. Additionally, 68 (26%) AYLH reported improved communication with HCWs, while 68 (20%) reported improved communication with caregivers. Regarding sexual behavior, 41 (15%) AYLH reported that disclosure helped them make the decision to abstain from sexual activity, and 34 (13%) reported that disclosure helped them practice safer sex after disclosure. Approximately half (124 [47%]) of the AYLH reported subsequently disclosing their HIV status to someone else.

### Factors associated with satisfaction with the disclosure process

Having a higher perception of support from the clinic was associated with higher satisfaction with the disclosure process (aMD: 0.19 (0.05–0.33), *p* = 0.010). Similarly, higher caregiver support before and after the disclosure was associated with higher satisfaction with the disclosure process (aMD: 0.19 [0.06–0.31], *p* = 0.004 and 0.17 [0.03–0.31], *p* = 0.020, respectively). AYLH who suspected they were HIV positive before they were disclosed to had lower satisfaction when compared to those who never suspected (aMD: −0.37 [−0.74 to (−0.01)], *p* = 0.047). Finally, compared to AYLH who preferred earlier disclosure, those who felt that they should never have been disclosed to tended to have lower satisfaction with the disclosure process (aMD: −1.02 [−1.76 to (−0.28)], *p* = 0.021). Age, sex, education level, duration of being on ART, duration since the disclosure, person who disclosed, disclosure readiness, and whether the disclosure was planned were not associated with satisfaction with disclosure (Table 3).

**Table 3:** Factors associated with satisfaction with disclosure.

Factor	Satisfaction (*N* = 229) Mean (SD)^1^	Mean difference (95%CI)	Age-adjusted mean difference (95%CI)	Value of *p* (from age-adjusted analysis)
*Age*				
10–14	3.17 (0.78)	Reference		
15–19	3.08 (0.89)	−0.09 (−0.33–0.16)		0.475
20–24	3.10 (0.82)	−0.07 (−0.39–0.26)		0.690
*Sex*				
Male	3.09 (0.79)	Reference	Reference	
Female	3.13 (0.87)	0.03 (−0.19–0.26)	0.04 (−0.25–0.32)	0.746
*Education level*				
Primary	3.14 (0.78)	Reference	Reference	
Secondary	3.08 (0.90)	−0.06 (−0.29–0.16)	−0.06 (−0.39–0.27)	0.663
Tertiary	3.22 (0.97)	0.08 (−0.49–0.66)	0.08 (−0.83–0.99)	0.821
*Duration on ART*				
<1 year	3.17 (0.41)	Reference	Reference	
1–4 years	3.06 (1.11)	−0.11 (−0.88–0.66)	−0.12 (−0.77–0.53)	0.664
≥5 years	3.13 (0.81)	−0.03 (−0.73–0.67)	−0.04 (−0.63–0.54)	0.850
*Duration since disclosure*				
<1 year	2.92 (0.90)	Reference	Reference	
1–4 years	3.03 (0.84)	0.12 (−0.41–0.64)	0.11 (−0.84–1.06)	0.747
≥5 years	3.15 (0.84)	0.24 (−0.26–0.73)	0.25 (−0.81–1.31)	0.547
Pre-disclosure clinic support*		**0.19 (0.08–0.30)**	**0.19 (0.05–0.33)**	**0.010**
Always	3.27 (0.81)			
Usually	2.93 (0.88)			
Sometimes	2.61 (0.88)			
Never	3.00 (0.69)			
Pre-disclosure caregiver support*		**0.18 (0.08–0–29)**	**0.19 (0.06–0.31)**	**0.004**
Always	3.25 (0.86)			
Usually	2.95 (0.78)			
Sometimes	2.78 (0.70)			
Never	2.79 (0.88)			
Post-disclosure clinic support*		0.14 (−0.02–0.30)	0.14 (−0.02–0.30)	0.158
A lot	3.15 (0.88)			
Some	3.11 (0.63)			
A little	3.00 (0.65)			
Not at all	1.5 (0.71)			
Post-disclosure caregiver support*		**0.17 (0.03–0.31)**	**0.17 (0.03–0.31)**	**0.020**
A lot	3.18 (0.85)			
Some	3.00 (0.52)			
A little	2.97 (0.78)			
Not at all	2.33 (1.21)			
Clinic prepared adolescent*		0.07 (−0.04–0.19)	0.07 (−0.11–0.26)	0.402
A lot	3.18 (0.82)			
Some	3.04 (0.89)			
A little	3.05 (0.82)			
Not at all	2.93 (1.1)			
*Suspected HIV-positive status*				
No	3.14 (0.86)	Reference	Reference	
Yes	2.77 (0.86)	**−0.37 (−0.71–[−0.03])**	**−0.37 (−0.74–[−0.01])**	**0.047**
Do not know	3.50 (0.55)	0.36 (−0.32–1.03)	0.36 (−1.35–2.06)	0.362
*Preferred time for disclosure*				
Earlier	3.15 (0.83)	Reference	Reference	
Later	3.35 (0.82)	0.20 (−0.10–0.50)	0.20 (−0.25–0.66)	0.282
Never	2.12 (0.99)	**−1.03 (−1.61–[−0.45])**	**−1.02 (−1.76–[−0.28])**	**0.021**
Do not know	3.05 (0.74)	−0.11 (−0.35–0.14)	−0.11 (−0.34–0.13)	0.373
*Was ready to be disclosed to*				
No	3.13 (0.92)	Reference	Reference	
Yes	3.12 (0.81)	−0.01 (−0.27–0.25)	−0.02 (−0.50–0.47)	0.930
Not sure	2.83 (1.17)	−0.30 (−1.01–0.41)	−0.30 (−4.25–3.64)	0.673
*Who disclosed HIV status*				
Caregiver	3.13 (0.77)	Reference	Reference	
Healthcare worker	3.10 (0.88)	−0.03 (−0.25–0.19)	−0.03 (−0.36–0.30)	0.814
Self-discovery	3.00 (1.31)	−0.13 (−0.73–0.48)	−0.12 (−0.92–0.67)	0.736
*Planned disclosure*				
Yes	3.15 (0.78)	Reference	Reference	
No	3.00 (0.82)	−0.03 (−0.32–0.26)	−0.03 (−0.45–0.39)	
Do not know	3.12 (0.95)	−0.15 (−0.47–0.17)	−0.15 (−0.63–0.32)	

^1^Satisfaction; range = 1–4. *Factor included in the linear regression model as a continuous variable. Bold: Significant association with satisfaction with the disclosure process.

## Discussion

In this treatment-experienced group of AYLH, the majority were adolescents who acquired HIV perinatally and had known their HIV status for a considerably long period of time. The majority preferred an early age of disclosure (12 years or less) and that a caregiver disclosed their HIV status. The majority were satisfied with the disclosure process and felt supported by the clinic and caregivers before and after the disclosure, while slightly over half reported being adequately prepared by the clinic. Clinic and caregiver support were associated with higher satisfaction with the disclosure process, while suspicion that one was living with HIV before disclosure was associated with lower satisfaction. This study builds on an established theoretical framework and previous research by including the perspectives of adolescents regarding factors that affect and the outcomes affected by the disclosure process.

Disclosure to children and adolescents about their HIV status is a process that culminates in full disclosure, i.e., the adolescent is aware and able to name HIV as the condition that they are living with (14). Preferences of age at full disclosure have been previously studied among 31 AYLH in Zimbabwe (21) and 37 AYLH in South Africa, and as in this study, AYLH preferred early disclosure (22). Healthcare workers also advocate for disclosure at an early age (before the age of 12 years), citing improved adherence and children’s right to know as major motivators. However, in previous studies, caregivers had favored disclosure when the child was older; for example, in Ethiopia, 50% were comfortable with disclosure before the age of 14, while the rest preferred disclosure at the ages of 15 and above (27–29). Fears expressed by caregivers about disclosure at an early age included social rejection, worry by the child, doubt whether the child will understand their HIV diagnosis, and difficulties of the child to keep their diagnosis a secret (27, 28).

In this study, slightly over half of the AYLH preferred caregivers to lead the disclosure process. The preference mirrored the current practice, where we reported that the majority of the AYLH were disclosed to by a caregiver. A few reported that they discovered their HIV status on their own, and though they did not elaborate on how the discovery happened, it may have included identifying the purpose of the clinic they visited (HIV clinic) or the medication they took. We found a higher preference for disclosure by caregivers among older adolescents and those with a longer duration since knowing their HIV status. These findings may be an indication of the evolution in the appreciation of the disclosure process and the HIV diagnosis that AYLH go through as they age (21). Disclosure practitioners and policymakers should appreciate that the AYLH’s preferences and the impact of disclosure could change as they grow older. The adolescent’s relationship with their caregivers could also determine their preferences. A previous qualitative study showed that adolescents preferred disclosure in a healthcare setting, where HCWs can provide accurate information and owing to the belief that HCWs would be more straightforward (21). The study, however, did not explore whether the AYLH wanted the caregivers present during the clinic sessions.

Many studies with HCWs and caregivers advocate for either the caregiver to take the leading role or to be actively involved together with an HCW (21), though caregivers have previously been reluctant to lead the process, citing a lack of skill on how to disclose appropriately (28). The caregivers, however, appreciate the benefits of disclosure on adherence and reported feeling relieved after the disclosure happened (9). There was no difference in satisfaction between AYLH disclosed to by a caregiver versus that by a HCW, which supports the practice of having both fully involved.

The preference of a majority of AYLH for full disclosure at an early age (≤12 years) and the involvement of caregivers in the disclosure process in this study are in agreement with current disclosure guidelines (14). Supporting caregivers to overcome their fears of early disclosure and interventions to increase their disclosure skills are necessary to ensure their meaningful involvement. We further demonstrated that the support AYLH received in the clinic and from caregivers during and after the disclosure was key to their satisfaction with the process, amplifying the need for broad, purposeful engagement of both during the disclosure process. Furthermore, starting the disclosure process early for adolescents receiving HIV care from childhood forestalls dissatisfaction when they suspect that they are living with HIV while this information is being hidden from them.

While preparation in the clinic was not associated with satisfaction with the disclosure experience, it has been shown in previous studies to improve post-disclosure outcomes (15, 16). In our study, only half of the participants felt that they were prepared adequately in the clinic. Furthermore, a quarter of AYLH were unequivocal that they were not ready for disclosure when it happened and that the disclosure should have been done later or never done. Future qualitative interviews should explore the perspectives of AYLH on what they feel would be the adequate preparation before disclosure. In the last 5 years, several tools to guide HCWs in preparing adolescents for disclosure in SSA have been found to be effective in increasing AYLH knowledge of HIV and in improving clinical outcomes (12, 15, 16). Other effective interventions such as “Amagugu” (30) and “Track” (31), though focused on the disclosure of a parent’s HIV status to their children, could be adapted for the disclosure of a child’s HIV status. However, these tools offer little guidance on caregiver roles, and their assessments rarely include implementation outcomes, including satisfaction by adolescents and caregivers with the disclosure process. Development and scale-up of disclosure tools targeting caregivers to increase their knowledge of HIV, adolescence, and the disclosure process as envisioned in disclosure guidelines (14) and assessing whether the tools improve adolescents’ disclosure experience are important next steps.

This study added to the body of knowledge of the potential benefits of disclosure, especially highlighting what AYLH felt were the important roles of disclosure. The majority felt that it had a positive influence on treatment adherence and clinic attendance, while a few mentioned that it improved their mood and sexual behavior. The current evidence is mixed on the impact of disclosure on the adherence to treatment (9, 32), mental health (33), and sexual behavior, which may impact onward HIV transmission (34).

The strength of this study is that it included the representation of a broad age range (12–24 years) of AYLH, with a wide geographical representation across counties in Kenya and HIV epidemic diversity. Limitations include the cross-sectional design of the study and recall limitations. Over two-thirds of our study population were disclosed to 5 years prior to this study or earlier. While the longer duration may have resulted in recall challenges, it also provided adequate time for the AYLH to experience and speak to the outcomes assessed, including the impact on communication, adherence, and sexual behaviors. Additionally, we did not establish the type of support provided by the clinic or caregivers and how preparation for the disclosure by the clinic was done. We also failed to collect the age at disclosure, an oversight during the development of the data collection tool. Finally, we did not use validated measures for some of the concepts, including support by clinic and caregivers and readiness for disclosure, with the questions asked therefore being an estimation of the adolescents’ perspectives.

## Conclusion

Young adults living with HIV advocated for an appropriately timed disclosure process involving caregivers and HCWs, which is aligned with the current disclosure guidelines. Support from caregivers and HCWs during and after disclosure is important for improving the disclosure experience.

## Data availability statement

The raw data supporting the conclusions of this article will be made available by the authors, without undue reservation.

## Ethics statement

The studies involving humans were approved by Kenyatta National Hospital Ethics and Research Committee. The studies were conducted in accordance with the local legislation and institutional requirements. The ethics committee/institutional review board waived the requirement of written informed consent for participation from the participants or the participants’ legal guardians/next of kin because the survey was considered low risk with participants only required to provide oral consent. Since no personal identifying information was collected from participants, a breach of confidentiality was of higher risk with written documentation rather than verbal agreement.

## Author contributions

CMu was responsible for conceptualizing the study and data analysis, writing the first draft, and overseeing revisions of the manuscript. GJ-S, DW, KB-S, IN, and CMu were involved in grant writing, development of the study protocol, and study tools and material. CMu, IN, CMb, AO, and JI were involved in data collection, and AO reviewed the analysis code. All authors contributed to the article and approved the submitted version.
